# News and Coming Events

**Published:** 1908-03-21

**Authors:** 


### NEWS AND COMING EVENTS.

Mr. Peyton Beale has been appointed surgeon to the ity Dispensary.

Dr. B. Hudson has been appointed assistant physician the Royal Hospital for Diseases of the Chest.

The annual general meeting of University College Hos- ital will be held on Thursday, March 26, at 4 o'clock.

The United Kingdom Hospitals Conference will be held t University College, London, on Wednesday and Thurs- ay, April 1 and 2.

Mr. John Goland, F.R.C.S., late senior surgeon to the ity Orthopædic Hospital, has resigned the post of surgeon the Royal National Orthopædic Hospital, to which he as recently elected.

Dr. H. Hyslop Thomson, formerly medical superinten- ent, Consumption Sanatorium of Scotland, Bridge of Veir, has been appointed resident medical officer to the iverpool Sanatorium, Kingswood, Delamere Forest.

The Therapeutical and Pharmacological section of the oyal Society of Medicine will meet at the Apothecaries' all, Blackfriars, E.C., on Tuesday, March 24, at 4.30 p.m., when Dr. John Milne Bramwell will read a paper on "Hypnotism."

The following appointments to the staff of the Hamp- tead General Hospital, which has been amalgamated with he North-Western Hospital, have been made: Mr. Edmund wen, Mr. J. Jackson Clarke, and Mr. W. H. Clayton- reene, surgeons to in-patients; Sir John Broadbent, M.D., nd Dr. G. A. Sutherland, physicians to in-patients.

The King and Queen have given their patronage to the ational Conference on Infantile Mortality, which will be eld this year at the Caxton Hall, Westminster, on March 24 nd 25, at 10 o'clock each day. Mr. Burns, M.P., the presi- ent, will deliver his presidential address on March 23, at p.m., following a reception of delegates at 8 p.m. The Mayor of Westminster will preside on March 24, and the ord Mayor of Liverpool on March 25.

It is proposed to hold an entertainment and fête in May, n the Clarence Wing of St. Mary's Hospital, Paddington, n aid of the institution. The area served by the hospital as been mapped out into centres. The Duchess of Rutland s president of the Marylebone centre, the Duchess of Suther- and of the St. George's, Hanover Square, centre, and the uchess of Abercorn of the centre for Westbourne Terrace. ady Dimsdale holds the office of chairman of the General adies' Committee.

The 68th Annual Meeting of the Governors of the Metro- olitan Convalescent Institution was held last week, Mr. M. O. FitzGerald in the chair. The number of patients admitted to the four Homes in 1907 was 7,515. Reference was made to the arrangements for the reception of patients requiring treatment after surgical operation in specially equipped wards at the Bexhill and Little Common Homes, with the object of relieving the great pressure upon the sur- gical beds in hospitals by receiving patients in an early stage of convalescence. The income of the institution in 1907 was £13,031, and the expenditure £14,228.

The Viscountess Esher has accepted the office of Vice-President of the Ladies' Committee of the Chelsea Hospital for Women.

Mr. A. E. Thomas, who for the past eight years has been Secretary of the Gravesend Hospital, has been appointed Assistant Secretary at the Royal Free Hospital.

Mr. Stanley Puckle and Miss Puckle have again given £1,000 for the endowment of a cot in the Tunbridge Wells General Hospital, making their total gifts to the institution £5,000 within three years.

Their Royal Highnesses the Prince and Princess of Wales and the Princess Mary on March 17 paid a surprise visit to the Hospital for Sick Children, Great Ormond Street, and spent about two hours going through the wards.

The Rev. A. W. Oxford, M.D., presided at the annual meeting of the Governors of the Samaritan Free Hospital for Women, Marylebone Road, on March 10. During the past year extensive building alterations and improvements were completed by the opening of a new operating theatre.

The trustees of the Prisons Charities have contributed 300 guineas to the Metropolitan Convalescent Institution, which has also received an anonymous donation of 1,000 guineas for the endowment of two beds, to be named "Pater" and "Mater," at the Bexhill Homes of the Institution.

The trustees of the Annie Zunz Trust have promised a contribution of £5,000 towards the rebuilding of the Royal National Orthopædic Hospital in Great Portland Street. The treasurers have already received the first instalment of £2,500. One of the wards of the new hospital is named the "Annie Zunz Ward."

Dr. Andrew Halliday Douglas, M.D., a former President of the Royal College of Physicians, Edinburgh, died recently, in his eighty-ninth year. He took the M.D. degree at Edinburgh in 1840, and became a member of the Royal College of Physicians, Edinburgh, in 1843. He had retired from practice, but had formerly been physician to the Royal Infirmary and to Chalmers' Hospital.

The Senate of the University of Glasgow has resolved to confer the honorary degree of doctor of laws (LL.D.) on Colonel David Bruce, C.B., M.B., C.M., F.R.S., D.Sc., R.A.M.C.; Mr. David McCowan, hon. treasurer of Glasgow Royal Infirmary; and John C. McVail, M.D., county medical officer, Stirlingshire and Dumbartonshire. These honorary degrees will be conferred in the Bute Hall on April 22.

At the annual meeting of the Central London Ophthalmic Hospital it was reported that on the recommendation of the Medical Committee a Teaching School for Ophthalmology had now been formed. Mr. Charles H. R. Wollaston, who presided, pointed out that the hospital had no endowments whatever and was entirely dependent on voluntary subscriptions. The lease of the hospital was rapidly running out, and it was absolutely necessary that before long they should take steps to consider where they could carry on the hospital in the future. To do this effectively a new hospital would have to be built, as the buildings were not at all suitable to present needs.

At the last meeting of the Council of the Royal College of Surgeons of England, Mr. W. A. Maggs, M.R.C.S., L.D.S., Dental Surgeon to Guy's Hospital, was re-elected a member of the Board of Examiners in Dental Surgery, and Mr. Rickman J. Godlee was appointed the representative of the College of Surgeons on the Council of University College, Bristol, in the vacancy caused by the resignation of Mr. Thomas Bryant.

The next meeting of the Odontological Section of the Royal Society of Medicine will take place on Monday, March 23, at 8 p.m., when Mr. Hopewell-Smith will exhibit and describe two specimens. Mr. Sturridge will show a case to demonstrate a point in the treatment of pyorrhœa, illustrated by the epidiascope. The discussion will be resumed on Mr. J. F. Colyer's paper, "The Treatment of Children from a Dental Aspect."

The Earl of Stamford presided on March 13 at the Annual Court of the Royal Free Hospital. There was a large attendance of Governors. The report shows that during the past year two new operating theatres have been erected, while a new mortuary chapel had been provided by the Ladies' Association, and a house has been acquired and furnished for the Maternity Department staff. Mr. Holroyd Chaplin, the Chairman of the Weekly Board, referred to the large proportion of the inhabitants of London who received relief in voluntary hospitals and other public institutions, and pointed out that the voluntary hospitals greatly relieved the rates; without them the accommodation of the Poor-law infirmaries would have to be largely increased.

The President of the Local Government Board has authorised for the current year the following researches, in addition to those already announced, under the grant voted by Parliament in aid of scientific investigations concerning the causes and processes of disease: (1) Further studies by Dr. F. W. Andrewes and Dr. T. J. Horder as to methods of inhibiting in the animal body the activities of infection by certain cocci; (2) a study of the various forms of pneumonia, especially in children, by Mr. A. G. R. Foulerton, F.R.C.S.; (3) a study of acid-fast bacilli in butter, by Dr. D. N. Nabarro; (4) an investigation of the injurious gases evolved during artificial illumination, by Dr. J. Wade, D.Sc.

The President of the Royal College of Surgeons has fixed July 1 as the date for closing the poll of the fellows and members on the subject of admitting women to the diplomas of the College. The following notice is to be circulated to fellows and members: "The Council, although they have decided that it is desirable to admit women to examination for the diploma of member, and although they have power to act upon this opinion, are anxious, in accordance with a resolution passed at a meeting of the Council on May 9, 1907, to obtain the views of the fellows and members on the matter. In regard to the corporate position of women, if the diplomas of the College be granted to them, the Council have taken legal opinion, and are advised that while under the Medical Act of 1876 women can be admitted as members or as fellows, no woman so admitted would thereby be entitled to take any part in the government, management, or proceedings of the College. (*a*) In your opinion, is it desirable that women should be admitted by examination as members of the College? (*b*) In your opinion, is it desirable that women, after admission to the membership, should be admitted by examination as fellows of the College?"

At the annual general meeting of the Governors of th[illegible] French Hospital and Dispensary in Shaftesbury Avenue hel[illegible] recently it was reported that 929 in-patients were receive[illegible] last year and 5,532 out-patients treated. Two hundred a[illegible] eighty convalescents, 36 of whom were children, were sent [illegible] the convalescent home at Brighton. The statistics quot[illegible] were eloquent of the cosmopolitan character of the hospit[illegible] nearly all French-speaking nationalities being represented [illegible] the patients; but even more convincing were the subscri[illegible]tions received from nearly every European country a[illegible] Southern States, with donations from King Edwar[illegible] Hospital Fund, the Hospital Saturday Fund, and t[illegible] Metropolitan Saturday Fund. At the fortieth annu[illegible] banquet, to be held on May 16, M. Paul Cambon w[illegible] preside and the Lord Mayor and Sheriffs will attend.

The Bishop of North Queensland (Dr. Frodsham), w[illegible] has lately come over to England, is preparing to ma[illegible] arrangements with the schools of tropical medicine in Eng[illegible]land to send out to Australia a trained man to start the wo[illegible] of the institute which it is proposed to form at Townsvil[illegible] in Northern Queensland, for the study of tropical disease[illegible] The general management will be undertaken by the thr[illegible] Australian Universities having medical schools—Sydne[illegible] Melbourne, and Adelaide. The Australian Governme[illegible] will subsidise the work at the rate of £450 per annum, a[illegible] the Government of Queensland will give £250; and the[illegible] sums will be increased by private subscriptions. T[illegible] managers of the Townsville Hospital will set aside a buil[illegible]ing for a laboratory free of charge. Correspondence relati[illegible] to the matter may be addressed to the Bishop at the Chur[illegible] House, Westminster.

